# Prognostic Bone Metastasis-Associated Immune-Related Genes Regulated by Transcription Factors in Mesothelioma

**DOI:** 10.1155/2022/9940566

**Published:** 2022-01-27

**Authors:** Zhiquan Hao, Siqiao Wang, Zixuan Zheng, Jiehan Li, Wanting Fu, Donglin Han, Yinrou Huang, Qing Lin, Shuyuan Xian, Penghui Yan, Man Li, Ruoyi Lin, Tong Meng, Jie Zhang, Zongqiang Huang

**Affiliations:** ^1^The First Clinical College, The First Affiliated Hospital, Jinan University, Guangzhou 510630, China; ^2^Institute of Orthopedic Diseases and Department of Bone and Joint Surgery, The First Affiliated Hospital, Jinan University, Guangzhou, Guangdong 510630, China; ^3^Department of Orthopedics, The First Affiliated Hospital of Zhengzhou University, 1 East Jianshe Road, Zhengzhou, China; ^4^Tongji University School of Medicine, 1239 Siping Road, Shanghai 200092, China; ^5^Department of Human Anatomy, School of Basic Medical Sciences, Fujian Medical University, Fuzhou 350122, China; ^6^Division of Spine, Department of Orthopedics, Tongji Hospital Affiliated to Tongji University School of Medicine, Shanghai 200065, China

## Abstract

Mesothelioma (MESO) is a mesothelial originate neoplasm with high morbidity and mortality. Despite advancement in technology, early diagnosis still lacks effectivity and is full of pitfalls. Approaches of cancer diagnosis and therapy utilizing immune biomarkers and transcription factors (TFs) have attracted more and more attention. But the molecular mechanism of these features in MESO bone metastasis has not been thoroughly studied. Utilizing high-throughput genome sequencing data and lists of specific gene subsets, we performed several data mining algorithm. Single-sample Gene Set Enrichment Analysis (ssGSEA) was applied to identify downstream immune cells. Potential pathways involved in MESO bone metastasis were identified using Gene Oncology (GO) analysis, Kyoto Encyclopedia of Genes and Genomes (KEGG) analysis, Gene Set Variation Analysis (GSVA), Gene Set Enrichment Analysis (GSEA), and Cox regression analysis. Ultimately, a model to help early diagnosis and to predict prognosis was constructed based on differentially expressed immune-related genes between bone metastatic and nonmetastatic MESO groups. In conclusion, immune-related gene SDC2, regulated by TFs TCF7L1 and POLR3D, had an important role on immune cell function and infiltration, providing novel biomarkers and therapeutic targets for metastatic MESO.

## 1. Introduction

Mesothelioma (MESO) is a rare neoplasm with high mortality. Pleural mesothelioma is the most common MESO, the main cause of which is asbestos deposition in the lung. Despite the reduction of asbestos use, the morbidity of MESO is still increasing because of the long latency period [1–3]. And the peak of MESO is predicted to come during 2012-2030, which varies in different location [4, 5].

Commonly, early diagnosis is an indicator of a good prognosis. However, diagnosis of MESO often occurs when clinical presentation appeared which indicates the late stage. Patients diagnosed in early stage are usually identified when examined by radiation test for other disease, and they indeed have favorable outcomes [6, 7]. Low efficiency of traditional therapeutic strategies also contributes to the malignancy of MESO [8]. Excitingly, biomarkers for early diagnosis of several cancers have shown promising application values [9–11]. Immunotherapy of MESO exhibits satisfactory clinical outcomes as well [12]. Bone metastasis is a rare but much more serious feature in MESO [13, 14]. Hence, it is of high clinical significance to elucidate immune-related molecular mechanisms that are associated with MESO bone metastasis, which will aid in the amelioration of individualized therapeutic methods for advanced patients.

Molecular signatures as well as tumor-associated cells have high predictive values for cancer outcomes [15]. The immunological data containing cell type, location, and density even show better predictive results than traditional histopathological methods [16]. Expression level of transcription factors (TFs) was associated with survival in various cancers [17–19]. Importantly, TFs have a regulatory function in cell differentiation especially in immune cells [20–22]. Previous studies have identified various biomarkers and their regulatory networks in MESO, but few studies have focused on immune-related genes and TFs [23–25]. Therefore, this study is innovative in immune-related MESO bone metastasis-related biomarkers and individualized therapeutic targets. The prognosis of bone metastatic MESO is expected to be improved by affecting immune-related signaling axes in this study.

Here, we constructed a model to forecast the prognosis based on differently expressed immune-related genes between the bone metastatic group and nonmetastatic group. And we figured out key prognostic immune-related genes in MESO which were regulated by TFs. Potential downstream pathways and immune cells were further extracted using functional enrichment analysis, Gene Set Variation Analysis (GSVA), Gene Set Enrichment Analysis (GSEA), single-sample Gene Set Enrichment Analysis (ssGSEA), and Cox regression methods. Finally, a network was instituted considering all features above. The differential expression level of immune-related gene and TFs was further validated by immunohistochemistry experiment in tissue samples from patients with mesothelioma. Assay for Transposase-Accessible Chromatin with high-throughput sequencing (ATAC-seq) analysis was performed to determine the direct regulatory pattern between these immune-related genes and TFs.

## 2. Method

### 2.1. Data Collection and Differential Expression Analysis between Bone Metastatic and Nonmetastatic MESO Samples

Transcriptome profiling and clinical information of 86 MESO samples were derived from The Cancer Genome Atlas (TCGA) database (https://portal.gdc.cancer.gov/). The list of immune-related signatures was downloaded from the ImmPort database (http://www.immport.org/), which involved 3718 genes. Transcription factor profiles were additionally retrieved from the Cistrome database (http://www.cistrome.org/) and included 318 genes.

After eliminating repetitive genes, 86 cases were divided into bone metastatic and nonmetastatic groups. The Wilcoxon signed-rank test was applied. Genes with a false discovery rate (FDR) *P* value < 0.05 and log2 (fold − change) > 1.0 or <-1.0 were defined as differently expressed, and heat map as well as volcano plot was depicted. The study was approved by the Ethics Committee of the First Affiliated Hospital of Zhengzhou University.

### 2.2. Construction of the Immune Prognostic Model

Overlapped genes between the DEGs and immune-related genes were singled out and plugged into univariate Cox regression to identify survival-related genes. Genes with a *P* value < 0.05 were defined as prognostic associated significantly. We subsequently performed multivariate Cox regression to evaluate the regression coefficients of genes and construct the prognostic model. The cutoff for MESO patients was defined as the median risk score classifying cases as low- and high-risk groups. The accuracy of the model was appraised using the area under the ROC curve (AUC). Kaplan-Meier survival analysis was also performed to assess the predictive ability. Scatter plot and expression heat map were depicted to show the correlation of risk score with the survival, intuitively.

### 2.3. Independence of the Immune Prognostic Model from Traditional Clinical Features

Among 86 MESO cases, 84 MESO cases were subjected to further analysis as they have complete clinical information, including survival information, age, gender, histologic grade, pathologic stage, and TNM stage. To verify whether the risk score of the immune prognostic model was independent among these clinical features, univariate and multivariate Cox regression analyses were performed. The results were shown in forest maps.

### 2.4. Correlation Analysis between TF and Immune-Related Genes

Differentially expressed TFs were identified based on differential expression analysis with ∣log2 FC | >1.0 and FDR value < 0.05. Pearson correlation analysis was conducted to assess the potential regulatory patterns between these transcription factors and prognostic immune-related genes. Connections with a correlation coefficient > 0.010 and *P* value < 0.050 were extracted.

### 2.5. Identification of Bone Metastasis-Related Immune Cells

In order to figure out the fraction of 22 kinds of immune cells, CIBERSORT algorithm was used. Pearson correlation analysis was subsequently conducted to evaluate the correlation between the immune cells and the TF-regulated immune-related genes. Immune cells with a *P* value < 0.050 were finally extracted. Moreover, single-sample GSEA (ssGSEA) was conducted to uncover the DEG-enriched immune cells in MESO.

### 2.6. Functional Enrichment Analysis

Firstly, GO and KEGG functional analyses were performed using R package “org.Hs.eg.db/” (http://www.bioconductor.org/packages/release/data/annotation/html/org.Hs.eg.db.html) packages in Bioconductor to detect the potential functions of DEGs identified. The results were shown in bubble diagrams. Secondly, GSVA algorithm was used to obtain the expression level of all genes in KEGG pathways. Univariate Cox analysis was subsequently conducted to identify pathways that significantly associated with overall survival. And the correlations of them with a single gene were evaluated using Pearson correlation analysis. Moreover, GSEA algorithm was also used to identify differently enriched pathways between bone metastatic and nonmetastatic MESO samples.

### 2.7. Construction of the Interaction and Correlation Network

Regulation pairs above were completely included in the regulation network which was plotted by Cytoscape (3.7.1) [26]. In the network, immune-related gene, TFs, immune cells, and pathways were, respectively, defined as pink rhombus, green arrows, purple ellipses, and yellow rectangles.

### 2.8. External Validation/Online Database Validation

To minimize bias, multiple databases including the CellMarker [27], GeneCards [28, 29], String [30], Gene Expression Profiling Interactive Analysis (GEPIA) [31], PROGgeneV2 [32], UALCAN [33], UCSC Treehouse Childhood Cancer Initiative, Kaplan-Meier plotter [34], LinkedOmics [35], cBioPortal [36], and Cancer Cell Line Encyclopedia (CCLE) [37] were used to evaluate gene and protein expression levels of key biomarkers at the tissue level.

### 2.9. Immunohistochemistry Validation

Paraffin-embedded sections of diagnostic biopsies collected from trial MESO patients (bone metastatic patients and nonmetastatic patients) and tumor sections were stained with antibodies for SDC2 (Abcam, ab205884) and TCF7L1 (Abcam, ab248495). Then, these slides were counterstained with haematoxylin. Frozen tumor sections were utilized for detecting expression level and subcellular location of SDC2 and TCF7L1 immunohistochemistry in tumors between bone metastatic MESO patients and nonmetastatic MESO patients. Negative controls of identical tumor tissue sections were utilized; hence, the primary antibodies were omitted. The conditions were utilized for staining with individual antibodies according to the protocol of the manufacturers.

### 2.10. ATAC-seq Validation

ATAC-seq refers to an impressively flexible, simple, and powerful technique to profile chromatin regions genome-wide, compared with traditional methods like functional assays or sequence conservation analyses [38].

ATAC-seq data of MESO samples were downloaded from TGCA project of chromatin accessibility landscape of primary human cancers (https://gdc.cancer.gov/about-data/publications/ATACseq-AWG), which were then utilized to explore the chromatin accessibility in specific locations of key TFs and immune-related genes [39]. Furthermore, the binding relationship was determined by comparing with control groups using Gviz package [40, 41].

### 2.11. Statistical Analysis

All statistical analysis was conducted by R version 3.5.1 (Institute for Statistics and Mathematics, Vienna, Austria; http://www.r-project.org/) (Package: impute, UpSetR, ggplot2, rms, glmnet, preprocessCore, forestplot, survminer, survivalROC, and beeswarm). Two-tailed *P* < 0.05 was regarded statistically significant.

## 3. Result

### 3.1. Identification of Significantly Differently Expressed Genes and Functional Analysis

**Figure 1:**
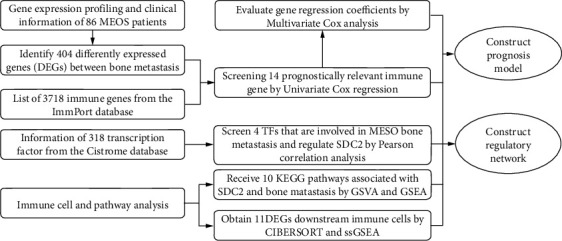
Flowchart of this study.

**Figure 2:**
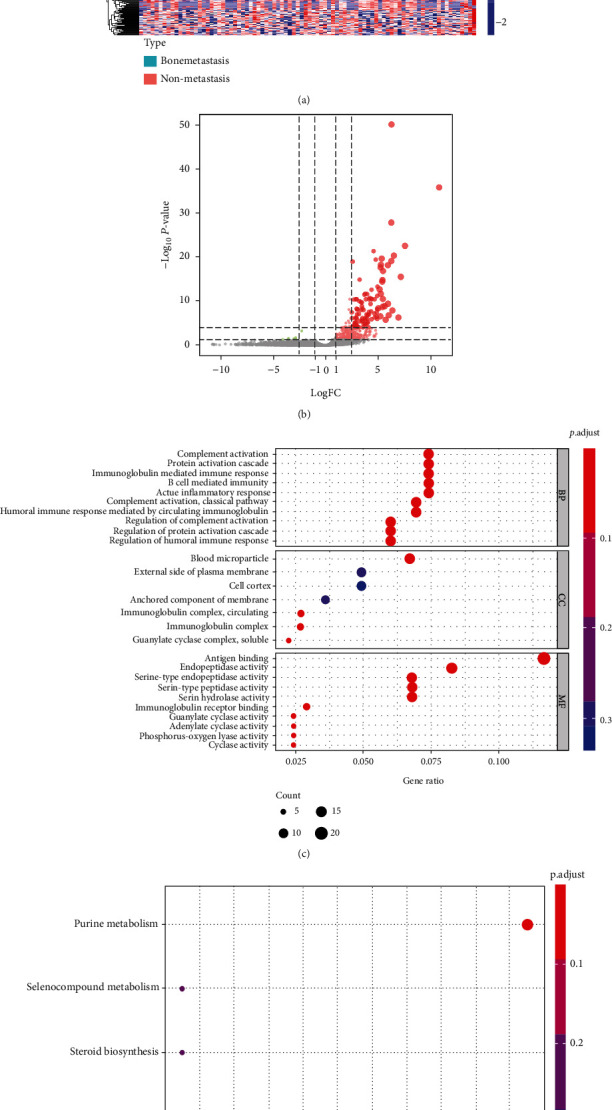
Different expressed genes in mesothelioma: (a) Expression of genes in MESO; (b) the volcano plot showed different expressed genes (DEGs) in MESO versus normal samples; (c, d) functional enrichment analysis of DEGs. Abbreviation: BP: biological process; CC: cellular component; MF: molecular function.

The flow diagram of this integrated analysis is shown in Figure 1. We obtained transcriptome profiles and clinical information of 87 MESO patients, consisting of 4 bone metastatic patients and 83 nonmetastatic patients, from TCGA database. 30960 genes were plugged into differently expressed analysis, and 404 genes, 396 upexpressed and 8 downexpressed, were finally identified as DEGs between bone metastatic and nonmetastatic groups (Figures 2(a) and 2(b)). Clinical information is summarized in Table 1.

### 3.2. GO and KEGG Functional Enrichment Analysis

DEGs were enriched in 27 GO items and 4 KEGG pathways (Figures 2(c) and 2(d)). GO items consisted of 10 biological processes (BP), 7 cellular components (CC), and 10 molecular functions (MF). Four KEGG pathways were “purine metabolism,” “selenocompound metabolism,” “steroid biosynthesis,” and “tryptophan metabolism.”

### 3.3. Construction of the Prognostic Model and Model Validation

**Figure 3:**
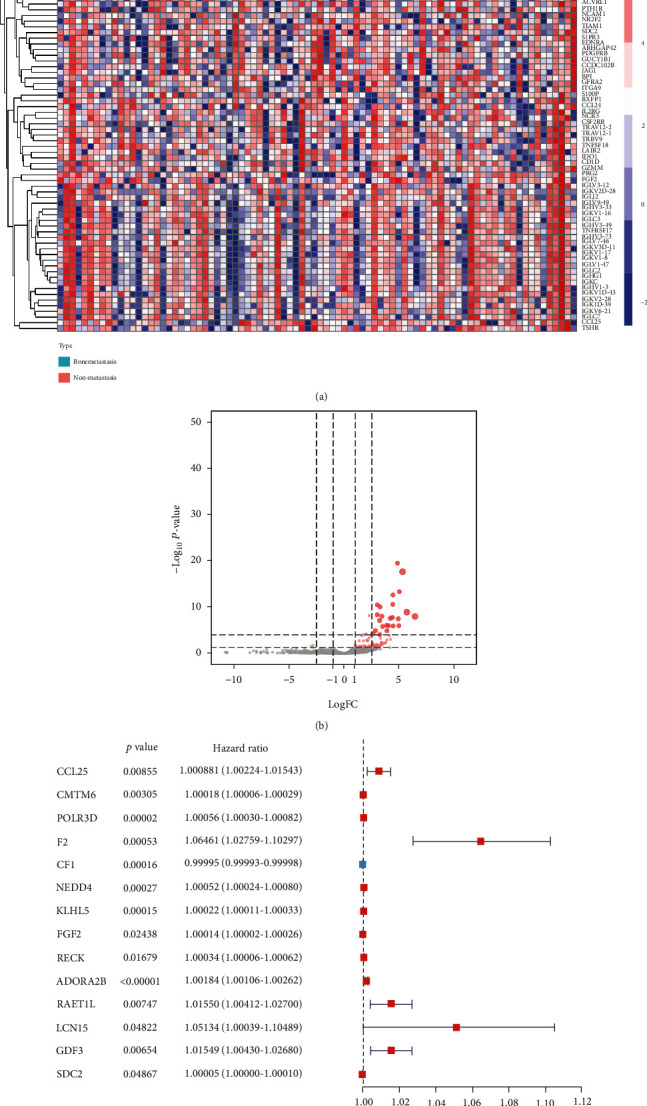
Filtration of key features in the prognostic model: (a) expression of immune-related genes from the ImmPort database in MESO; (b) volcano plot was drawn to show different expressed immune-related genes; (c) 14 immune-related genes were identified as prognosis associated using univariate Cox regression.

Gene expression profiles of 3718 immune-related genes were obtained from ImmPort database, and 78 immune-related genes were DEGs in MESO (Figures 3(a) and 3(b)). Univariate Cox regression was conducted, and 14 genes were significantly identified as prognosis associated (Figure 3(c)).

**Figure 4:**
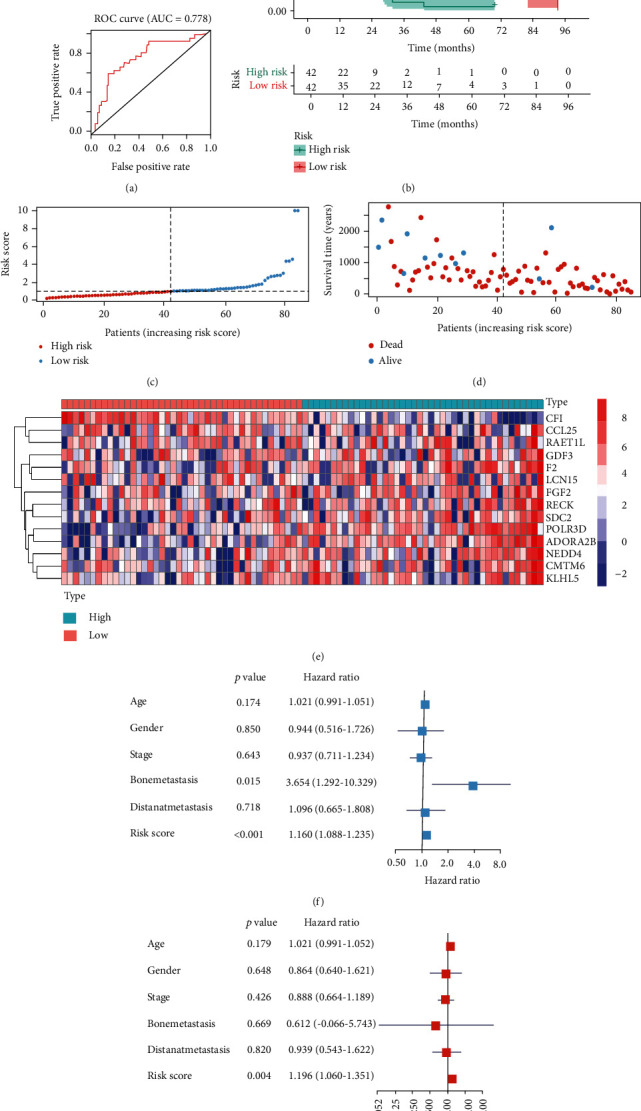
Model validation and independence of the predict model from traditional clinical features: (a) the high AUC (0.778) of the ROC curve indicating good predict power of the model; (b) overall survival of patients with MESO according to risk scores of the model; (c, d) survival status and risk score of 84 patients; (e) expression of key features in MESO patients; (f) bone metastasis and risk score were negatively, respectively, and significantly associated with prognosis using univariate Cox regression model; (g) risk score was negatively, respectively, and significantly associated with prognosis using the multivariate Cox regression model.

The AUC of the ROC curve was 0.778, indicating good predictive power (Figure 4(a)). Patients with high risk score revealed poor prognostic in Kaplan-Meier analysis (Figure 4(b)). Scatter plots showed the risk score and survival status of 84 patients with MESO (Figures 4(c) and 4(d)). Red circles enriched in the lower right corner in Figure 4(d) represent a good reliability of the model. Expression level of 14 features of the model was shown in the heat map (Figure 4(e)).

### 3.4. Independence of the Prognostic Model from Traditional Clinical Features

Univariate Cox regression analysis indicated that bone metastasis (HR = 3.65, *P* = 0.020) and a risk score (HR = 1.16, *P* < 0.001) were independent risk factors for prognosis in MESO (Figure 4(f)). Multivariate Cox regression analysis also proved the risk score was a risk factor for MESO independently (Figure 4(g)).

### 3.5. Regulation between Immune-Related Genes and TFs

**Figure 5:**
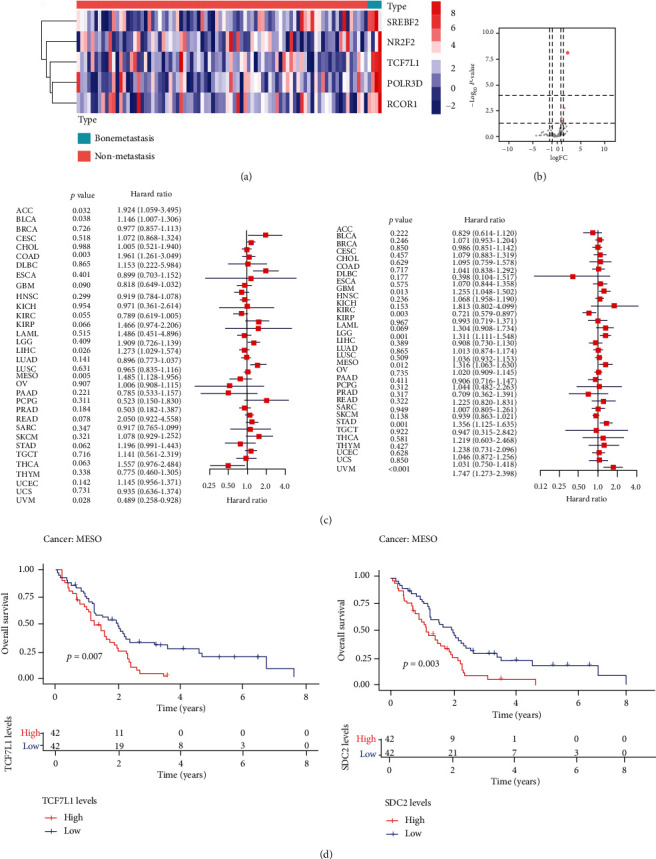
Identification of key transcription factors (TFs) in MESO: (a) expression of differently expressed TFs in patients with MESO; (b) volcano plot showed that 5 out of 318 TFs from the Cistrome database were differently expressed in MESO versus normal samples; (c) survival analysis of TCF7L1 (left) and SDC2 (right) in pancancer; (d) effect of expression levels of TCF7L1 and SDC2 on the survival status of patients with MESO.

318 TFs were derived from the Cistrome database, and 5 TFs (SREBF2, NR2F2, TCF7L1, POLR3D, and RCOR1) were identified as differentially expressed TFs involved in MESO bone metastasis (Figures 5(a) and 5(b)). Based on the results of the Pearson correlation analysis, a total of four TFs (RCOR1, TCF7L1, POLR3D, and NR2F2) were coregulators of the same immune-related gene SDC2. Then, we performed survival analysis of TCF7L1 (left) and SDC2 (right) in pancancer, and the results are shown in the forest plot (Figure 5(c)). Kaplan-Meier analysis was performed to show the effect of expression levels of TCF7L1 and SDC2 on the survival status of patients with MESO (Figure 5(d)).

### 3.6. Identification of Bone Metastasis-Related Immune Cells

**Figure 6:**
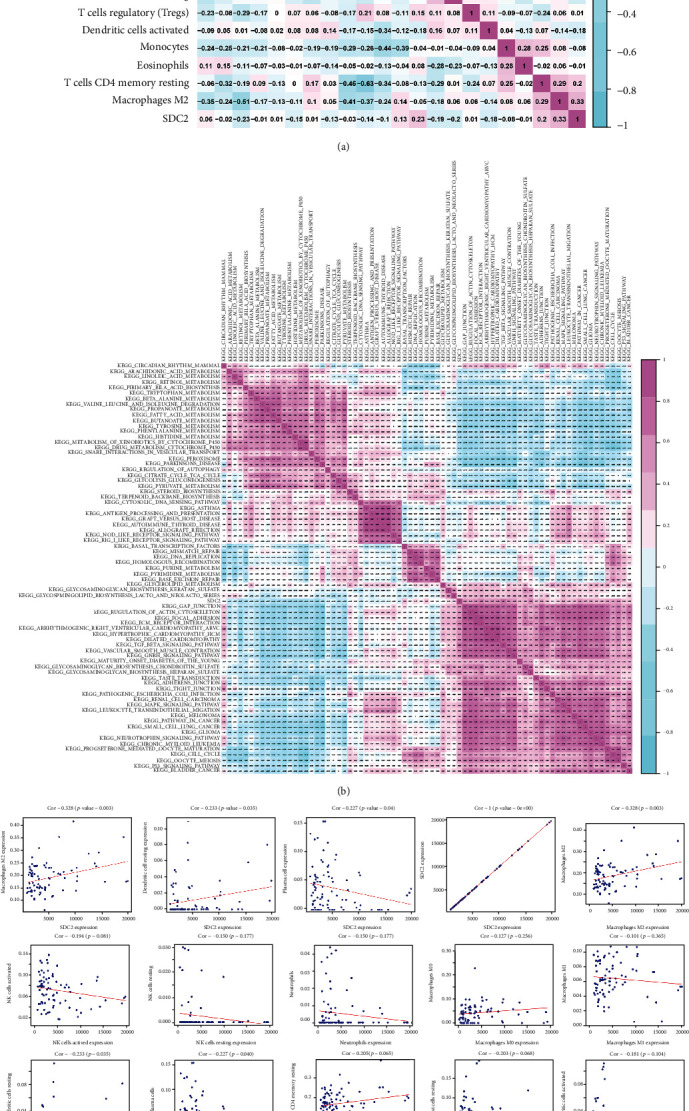
Coexpression of SDC2: (a) correlation between SDC2 and immune cells; (b) correlation between SDC2 and overall survival associated pathways. “TGF beta signaling pathway,” “ECM receptor interaction,” and “glycosaminoglycan biosynthesis heparan” were the top 3 SDC2-correlated KEGG pathways; (c) correlation of specific immune cells with SDC2. M2, dendritic resting cells and plasma cells were significantly associated with SDC2.

CIBERSORT algorithm was used to evaluate the fraction of immune cells. Coexpression analysis was conducted to evaluate the correlation of SDC2 with immune cells (Figure 6(a)). For M2, dendritic resting cells and plasma cells were significantly associated with SDC2 (Figure 6(c)). According to results of ssGSEA, 8 immune cells were identified as downstream cells of DEGs. Finally, 11 immune cells were extracted: plasma cells, macrophages M2, dendritic cells resting, cytolytic activity, DCs, iDCs, MHC class I, NK cells, parainflammation, type-I FN response, and type II IFN response.

### 3.7. Functional and Coexpression Analyses

According to the GSVA algorithm, 74 KEGG pathways were identified as significantly associated with overall survival (OS). The Pearson correlation analysis subsequently filtered 52 pathways that were significantly correlated with SDC2 (Figure 6(b)).

**Figure 7:**
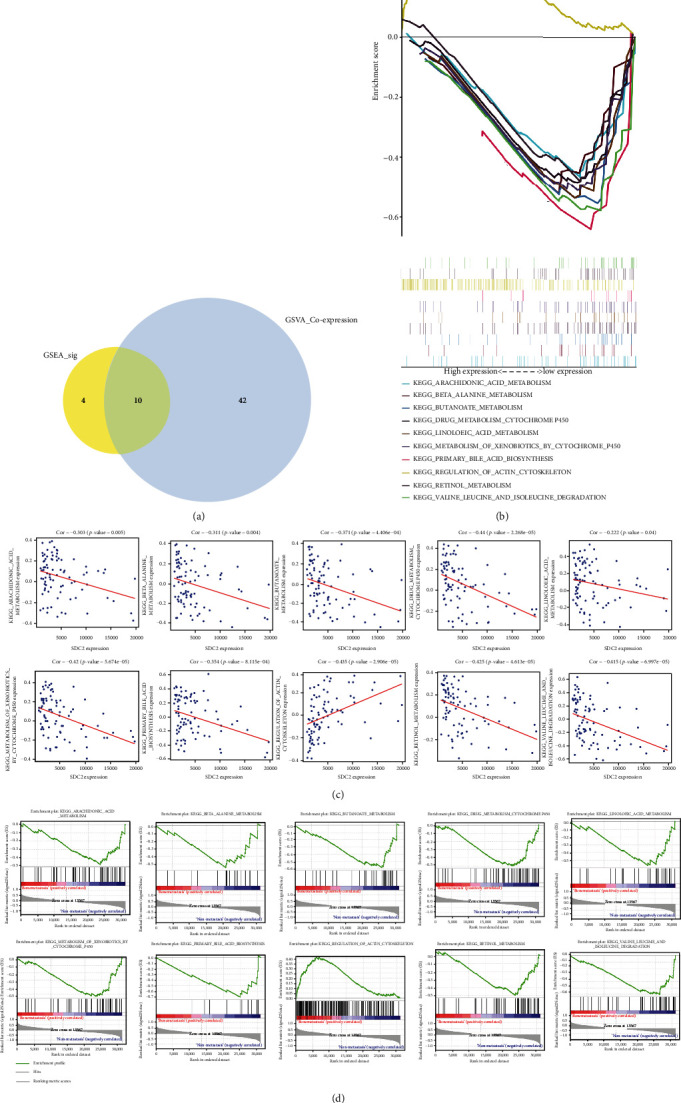
Filtration of relevant pathways: (a) 52 pathways correlated with SDC2 were figured out using Pearson correlation analysis; 14 pathways were identified as bone metastasis associated using GSEA algorithm; and 10 were overlapped; (b) summarizations of GSEA results; (c) results of 10 pathways in Pearson correlation analysis; (d) results of specific pathways using GSEA algorithm.

According to GSEA algorithm, 14 KEGG pathways were identified as differently enriched between bone metastatic and nonmetastatic groups. After coexpression analysis, 10 pathways were finally extracted (Figure 7(a)). Matched-rank GSEA results are shown in Figures 7(b) and 7(c). These pathways were “metabolism of xenobiotics by cytochrome P450,” “butanoate metabolism,” “primary bile acid biosynthesis,” “linoleic acid metabolism,” “beta alanine metabolism,” “retinol metabolism,” “arachidonic acid metabolism,” “valine leucine and isoleucine degradation,” “drug metabolism cytochrome P450,” and “regulation of actin cytoskeleton” (Figure 7(d)).

### 3.8. Construction of the Regulatory Network

**Figure 8:**
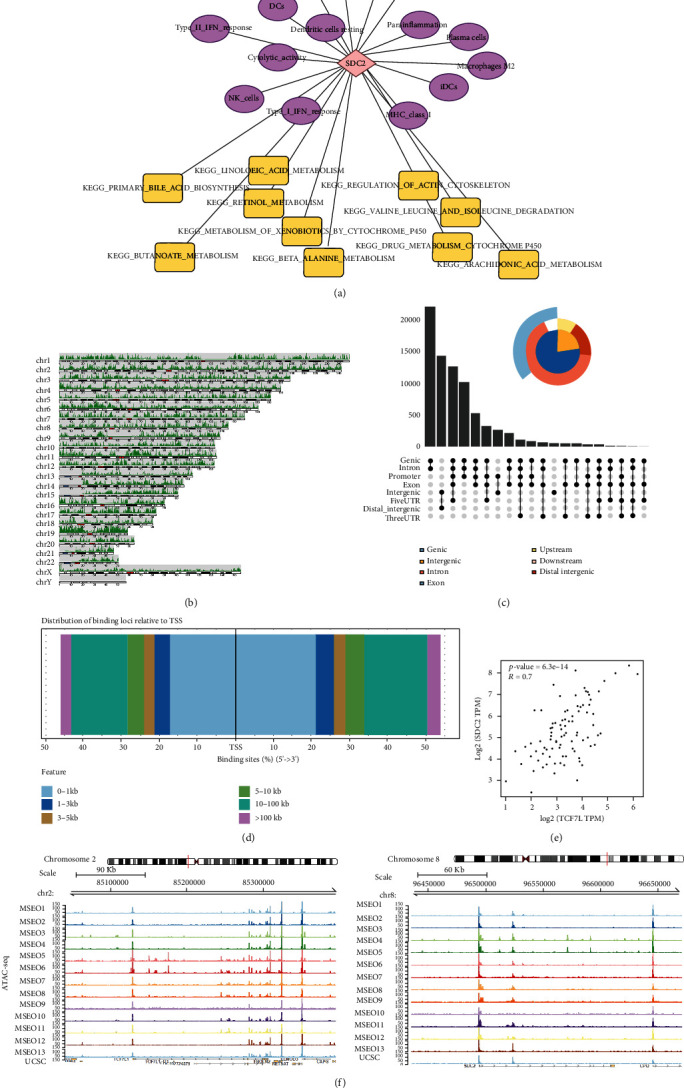
Immune regulatory network and ATAC-seq validation. (a) Integrated network includingSDC2 and 4 TFs, 11 immune cells, and 10 pathways; (b) gene loci on different chromosomes; (c) intersection of different pick types (genic, intergenic, exon, upstream, intron, and distal intergenic); (d) distribution of binding loci relative to TSS; (e) correlation analysis of TCF7L1 and SDC2 (*P* < 0.001, *R* = 0.700); (f) in ATAC-seq data of MESO samples, multiple binding peaks were identified in SDC2 and TCF7L1 sequences.

Integrated network is shown in Figure 8(a): SDC2 was the hub molecular of the network; SDC2 was regulated by 4 TFs (RCOR1, TCF7L1, POLR3D, and NR2F2) and had a function in 11 bone metastasis-associated immune cells (M2, dendritic resting cells, plasma cells, cytolytic activity, DCs, iDCs, MHC class I, NK cells, parainflammation, type-I FN response and type II IFN response) through 10 pathways (“metabolism of xenobiotics by cytochrome P450,” “butanoate metabolism,” “primary bile acid biosynthesis,” “linoleic acid metabolism,” “beta alanine metabolism,” “retinol metabolism,” “arachidonic acid metabolism,” “valine leucine and isoleucine degradation,” “drug metabolism cytochrome P450,” and “regulation of actin cytoskeleton.” Moreover, the results of external validation and potential effect of plasma cells (“butanoate metabolism” and “regulation of actin cytoskeleton”) are considered (Figure 8(a)).

### 3.9. Chromatin Accessibility Mapping Based on ATAC-seq Validation

Multiple open chromatin regions of SDC2 and TCF7L1 in sorted MESO cells were identified using ATAC-seq analysis. Open chromatin loci on different chromosomes (Figure 8(b)) as well as the distribution of binding loci relative to TSS were visualized, and the upsetplot showed the intersection of different pick types (genic, intergenic, exon, upstream, intron, and distal intergenic) (Figures 8(c) and 8(d)). Moreover, we analyzed the correlation between TCF7L1 and SDC2, and the results showed that the expression of TCF7L1 was positively correlated with SDC2 (*P* < 0.001, *R* = 0.700) (Figure 8(e)). There were strong ATAC-seq binding peaks in MESO cells at promoters of SDC2 and TCF7L1 and at various regulatory elements' binding areas in the introns and in introns of neighboring genes, which indicated these regions may function as potential regulatory elements on upstream of SDC2 and TCF7L1 sequences (Figure 8(f)).

### 3.10. High SDC2 and TCF7L1 Expression by MESO Cells Is an Indicator of Bone Metastasis and Poor Prognosis

**Figure 9:**
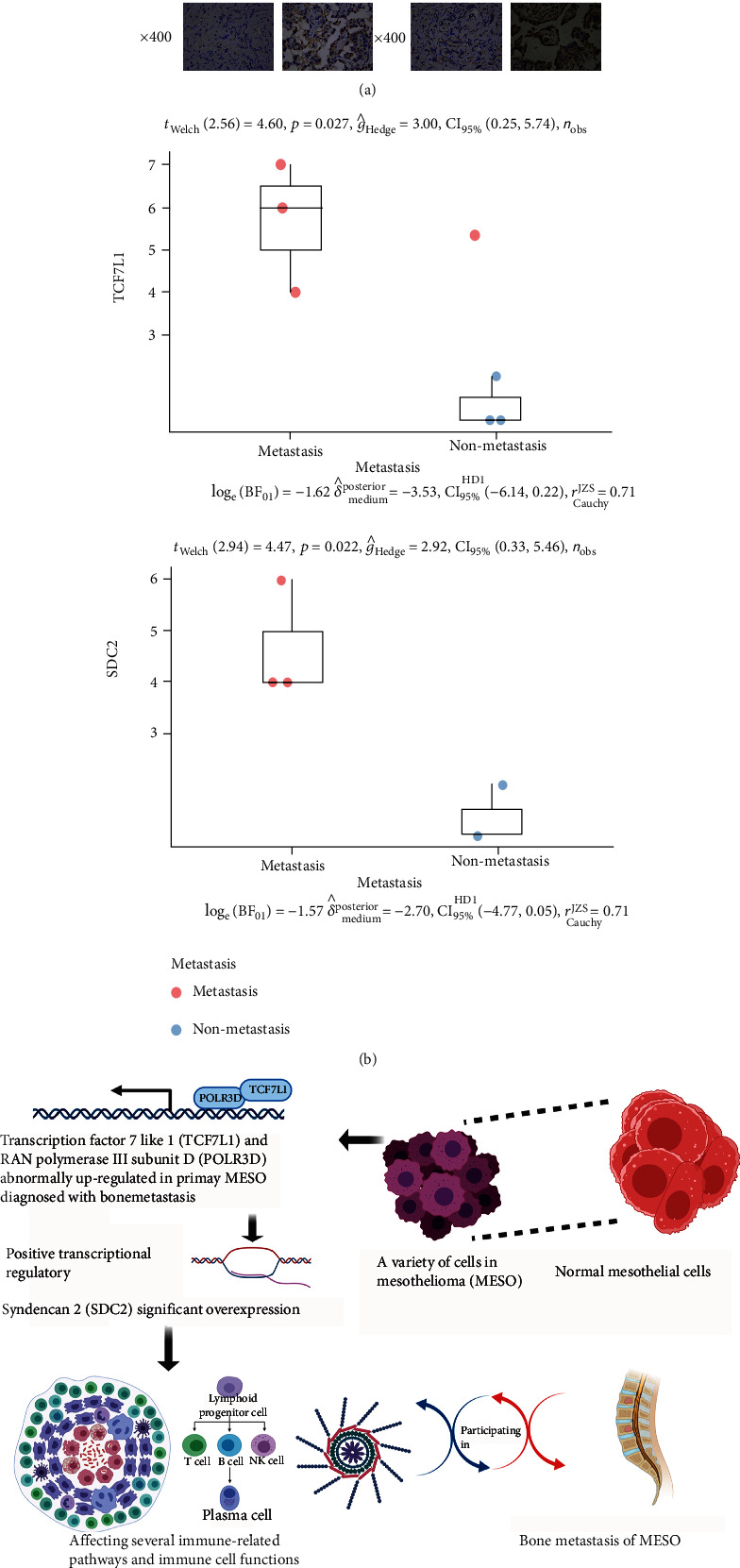
Immunohistochemical analysis of SDC2 and TCF7L1 expression in MESO biopsies: (a) immunohistochemical analysis of SDC2 and TCF7L1 expression in MESO specimens showing relatively higher expression in bone metastatic MESO samples; (b) expression level of SDC2 and TCF7L1 was significantly higher in bone metastatic MESO samples than that in nonmetastatic MESO samples byWelch's *t*-test (*P* < 0.05); (c) molecular mechanism of TCF7L1, SDC2, and immune features in MESO.

Based on the above, the expression level of key immune-related gene SDC2 and TF TCF7L1 was relatively higher in bone metastatic MESO samples than that in nonmetastatic MESO samples, which was significantly related to poor prognosis in MESO. However, there was no published information as to the source of this aberrant expression of SDC2 and TCF7L1 in tumor biopsies from patients with MESO. Hence, we stained for SDC2 (3 bone metastatic samples and 2 nonmetastatic samples) and TCF7L1 (3 bone metastatic samples and 3 nonmetastatic samples) in MESO pathological sections and also looked for any correlations with bone metastasis. Importantly, immunohistochemical staining of SDC2 and TCF7L1 in metastatic MESO tissue was more intense than that in nonmetastatic mesothelioma tissue, and this difference reached statistical significance (*P* < 0.05, Welch's *t*-test) (Figures 9(a) and 9(b)). The whole mechanism is shown in Figure 9(c) vividly.

### 3.11. External Validation

Firstly, we mined out all available markers of 11 immune cells using the CellMarker website. Top 5 key genes of 10 pathways were also found using the GeneCards database. Then, together with SDC2 and 4 TFs in the network, 60 biomarkers were finally input into several databases to test and verify our conclusion, which are summarized in Table S1. The overall correlation of 60 biomarkers was assessed using the String database and is shown in Figure S1A. And results of 19 most significant biomarkers out of 60 from other databases were finally selected and are shown as summarized in Table 2.

SDC2, the hub immune-related gene, was proved to be differently expressed gene in various cancers using the UALCAN database (Figure S1B). And it was significantly associated with OS in GEPIA (*P* = 0.001, Figure S1C), UCSC (*P* = 0.008, Figure S1D), and ProgGeneV2 (*P* = 0.016, Figure S1E). The transcription factor TCF7L1 was significantly correlated with OS in GEPIA (*P* = 0.009, Figure S1F), UCSC (*P* = 0.007, Figure S1G), and ProgGeneV2 (*P* = 0.003, Figure S1H). Another TF POLR3D was associated with OS significantly in GEPIA (*P* < 0.001, Figure S1I), UCSC (*P* < 0.001, Figure S1J), and UALCAN (*P* = 0.017, Figure S1K). CD38, the cell marker of plasma cells, was associated with OS in in UCSC (*P* = 0.045, Figure S2A) and GEPIA (*P* = 0.019, Figure S2B) and was correlated with distant metastasis in LinkedOmics (*P* = 0.018, Figure S2C). CD1A, cell marker of dendritic cells, DCs and iDCs, was correlated with OS in UCSC (*P* = 0.047, Figure S2D) and UALCAN (*P* = 0.007, Figure S2E).

As regards pathway analysis, there were at least two key genes of these pathways identified as OS-associated biomarkers which indeed confirmed our conclusion. Most significant genes were depicted, and details were as follows: CYP3A4 (*P* < 0.001, Figure S3A), ACAT2 (*P* < 0.001, Figure S3B), CYP27A1 (*P* = 0.007, Figure S3C), PLB1 (*P* = 0.003, Figure S3D), DPYS (*P* = 0.004, Figure S3E), GAD1 (*P* = 0.048, Figure S3F), LTC4S (*P* = 0.020, Figure S3G), PTGS1 (*P* = 0.024, Figure S3H), BCKDHB (*P* = 0.039, Figure S3I), ACTG1 (*P* = 0.028, Figure S3J), and ACTN1 (*P* < 0.001, Figure S3K) were associated with OS on tissue level in GEPIA. In ProgGeneV2, HMGCL (*P* = 0.013, Figure S4A), ACAT2 (*P* = 0.011, Figure S4B), CYP27A1 (*P* = 0.002, Figure S4C), PLB1 (*P* = 0.002, Figure S4D), GAD1 (*P* = 0.022, Figure S4E), LTC4S (*P* = 0.008, Figure S4F), PTGS1 (*P* < 0.001, Figure S4G), BCKDHA (*P* = 0.040, Figure S4H), ACTG1 (*P* = 0.049, Figure S4I), and ACTN1 (*P* < 0.001, Figure S4J) were significantly associated with OS. In UALCAN, CYP3A4 (*P* = 0.003, Figure S5A), ACAT2 (*P* = 0.010, Figure S5B), CYP27A1 (*P* = 0.016, Figure S5C), PLB1 (*P* = 0.004, Figure S5D), DPYS (*P* = 0.005, Figure S5E), GAD1 (*P* = 0.039, Figure S5F), BCAT1 (*P* = 0.005, Figure S5G), ACTG1 (*P* = 0.014, Figure H), and ACTN1 (*P* = 0.002, Figure S5I) were correlated with OS significantly. In LinkedOmics, genes associated with favorable (Figure S6A) and adverse (Figure S6B) OS are shown and summarized in the volcano plot (Figure S6C). HMGCL (*P* = 0.048, Figure S6D) and LTC4S (*P* = 0.024, Figure S6E) and BCKDHB (*P* < 0.001, Figure S6F) were significantly associated with OS. Moreover, results of LinkedOmics also indicated that HMGCL (*P* = 0.002, Figure S6G) and ACTN1 (*P* = 0.033, Figure S6H) were confirmed to be associated with distant metastasis. In UCSC, CYP3A4 (*P* = 0.005, Figure S7A), HMGCL (*P* = 0.010, Figure S7B), ACAT2 (*P* = 0.046, Figure S7C), CYP27A1 (*P* = 0.005, Figure S7D), PLB1 (*P* < 0.001, Figure S7E), DPYS (*P* = 0.015, Figure S7F), LTC4S (*P* = 0.039, Figure S7G), PTGS1 (*P* = 0.002, Figure S7H), and ACTN1 (*P* < 0.001, Figure S7I) were significantly associated with OS. Gene modification information of key factors was derived from cBioPortal (Figure S8A). Expression level of key genes in pleura was shown using data from the CCLE database (Figure S8B).

So we speculated that plasma cells, “butanoate metabolism” and “regulation of actin cytoskeleton” pathways, may be the most essential characters of MESO metastasis.

## 4. Discussion

MESO is mesothelial-derived neoplasm, and a peak morbidity was forecasted in the near future [4, 5]. Early diagnosis of mesothelioma is filled with challenges and pitfalls because of high expenses of routine radiological examination and morphology similarities with other diseases [42]. Routine treatment strategy of MESO is pemetrexed and platinum compounds, but no standard second-line strategy is proposed. Single-modality and multimodality treatment strategies of surgery, chemotherapy, and radiotherapy are discussed widely but no powerful conclusion has been raised so far [43]. These all enhance the mortality of MESO. Promisingly, several markers have been reported in MESO, though only few of them have shown high diagnostic specificity [44]. And MESO patients with specific infiltrating immune cells were reported to have a favorable prognosis after traditional treatment [45]. Various target therapy and immunotherapy approaches were also under investigation and dramatic effect in some cases indicated them to be hopeful candidates in the recent future [12]. Thus, more potential mechanisms considering immune features in MESO need to be clarified.

In this study, we figured out bone metastasis-related immune-related genes in MESO and constructed a prognostic model based on them. High AUC values and statistical significance in Cox regression analysis indicated good predictive power of the model. Additionally, we proposed a molecular mechanism that immune-related gene SDC2, regulated by TFs TCF7L1 and POLR3D, had a significant impact on the function and infiltration of immune cells and subsequently affected bone metastasis and prognosis of MESO (Figure S1). High expression level of SDC2 and TCF7L1 in bone metastatic MESO samples was validated by immunohistochemistry experiment, and ATAC-seq analysis of SDC2 and TCF7L1 demonstrated the direct transcriptional regulatory relationship between them. Among these immune cells, the correlation of plasma cells, dendritic cells, DCs, and iDCs with MESO survival was verified using external databases (Figure S2). Additionally, key features of 10 corresponding pathways were identified as survival or metastasis correlated using various databases (Figure S3, Figure S4, Figure S5, Figure S6, and Figure S7).

SDC2, a member of syndecan family, was demonstrated to participate in various cellular processes, such as cell proliferation, differentiation, apoptosis, cell adhesion, migration, and cytoskeletal organization [46–48]. On the tissue level, SDC2 was essential for tissue development, angiogenesis, cell communication, and modulation of microenvironment [49, 50]. Interestingly, binary effect of SDC2 was found in different tumors which correlated with the tissue origin and cancer subtypes. It had a cancer-promoting effect in epithelial tumors while shows a tumor-type response in mesenchymal cancers [51]. SDC2 was highly expressed in colon cancer [52], breast cancer [53], and glioma tissues [54] while generally not expressed in matched normal tissues. In colon cancer, SDC2 was significantly associated with tumor growth, cell migration, tumor stage, lymph and distant metastasis, and vascular invasion [52, 55]. Oh et al. demonstrated that quantification of SDC2 methylation could be a biomarker for early diagnosis of colorectal cancer (CRC) with a sensitivity of 90.0% and a specificity of 90.9% [10]. SDC2 could regulate cell migration and angiogenesis in cancer [56–58]. And both angiogenesis and antiangiogenic functions were reported. It also had an oncogenic function via epithelial-mesenchymal transition (EMT) [55]. As MET-targeted immunotherapy in MESO showed the effectiveness and safety in mice, potential mechanisms of SDC2 in MESO need to be clarified [59].

TCF7L1 is a downstream effector of the Wnt signaling pathway. Our analysis indicated that TCF7L1 might have a regulatory function on immune-related gene SDC2; in this way, it could impact metastasis and prognosis of MESO. Previous studies proved that it has a tumor-promoting role in multiple aggressive cancers [60–62]. The regulatory mechanism of TCF7L1 on tumor functional genes has been illuminated as well [60, 63]. Here, we uncovered the potential involvement of TCF7L1 in cancer metastasis, especially bone metastasis, which was not widely discussed before.

Function of immune cells in MESO has been studied before. MESO patients with CD8+ lymphocytes infiltrating showed a better prognosis after surgical treatment [45]. A significant amount of regulatory T cells were detected in mesothelioma tissues, and depletion of CD25+ T cells expressed an enhancement in survival in vivo [64]. The chimeric antigen receptor (CAR) T cell immunotherapies exerted promising treatment effects in vivo which indicated the therapeutic value of immune cells [59]. However, other immune cells were not clearly studied in MESO. Here, we detected potential regulation of plasma cells, dendritic cells, DCs, and iDCs in bone metastasis and prognosis in MESO.

Plasma cells are terminal functional status of B cell lineage and synthesize protective antibodies [65, 66]. It is a key factor of multiple myeloma and also participates in solid tumor progression [67]. Positive prognostic effect of plasma cells was affirmed in colorectal cancer, gastric cancer, esophageal cancer, and melanoma [68–71]. Negative prognostic effect was found in ovarian and breast cancer [72, 73]. Immunotherapies based on cytotoxic T cells showed a satisfactory effect on tumor treatment [74]. It was demonstrated that plasma cells have an immunosuppressive effect and it can impede T cell- dependent immunotherapy by inducing cell death [75]. Additionally, plasma cells could enhance the effectivity of prodrugs in colorectal cancer by secreting carboxylesterase [76]. In malignant mesothelioma (MPM), the localization of c-mesenchymal-epithelial transition (c-MET) on plasma membrane indicated longer survival of patients [77]. In our study, we uncovered the association between plasma cells and bone metastasis in MESO.

Dendritic cells (DCs) are the most powerful antigen-presenting cells (APCs) functioning in adaptive immune system [78]. There are several subsets of DCs, and iDCs represent the inflammatory DCs which functioned in antigen representation, migration, tumor rejection, and inducing antitumor responses [79]. DCs function by receiving and sending cell factors to regulate immune microenvironment and to influence cancer immunity [80]. In breast cancer, IL-10 secreted by macrophages could suppress interleukin- (IL-) 12 expressed in DCs and subsequently decrease pathologic complete response of cancer [81]. The antitumor function of DCs was dependent on T cell activation [82]. Density of mature DCs in non-small-cell lung cancer (NSCLC) was positively associated with density of T cells and prognosis of patients [83]. Binary functions of DCs were detected in ovarian cancer that at early stages, DCs has an antitumor effect while at advanced stages, it is a key factor of immunosuppression. This functional change resulted from a phenotype switching during cancer progression [84]. Dendritic cell vaccines have been brought into clinical trials. Though therapeutic effects were shown in some patients, there were still an extensive portion of patients who cannot obtain durable reactions. Thus, a more detailed commendation should be brought out [85]. Besides this, the importance of DCs in checkpoint therapy was also discussed. CD38 is a cell marker of DCs which is recorded in the CellMarker database [27]. It was reported that tumors escape from PD-1/PD-L1 blockade therapy through CD38-mediated immunosuppression [86]. It was demonstrated that expression of CD38 was enhanced after PD-1/PD-L1 blockade and a suppressive effect on CD8+ T cells was subsequently detected. Moreover, blockade of PD-L1 and CD38 simultaneously can improve the antitumor responses [86, 87]. In our study, the correlation of DCs with MESO prognosis was also confirmed and the potential regulatory mechanism of DCs regulation may help improve the treatment of MESO in the future.

To the best of our knowledge, this is the first study figuring out predictive biomarkers of MESO based on differently expressed immune-related genes between bone metastatic and nonmetastatic groups.

We also firstly elaborated on a regulatory mechanism considering immune-related genes, TFs, immune cells, and specific pathways. Nevertheless, there were still some limitations to our study. Firstly, our data were downloaded from public database; there were still some inexact records. And sample size of MESO in TCGA was relatively small. Secondly, our analysis mostly focused on mathematics. Thirdly, our cases were from western countries and caution should be observed when applying our conclusion to patients from other sources. More verification on tissue and cell level should be put into effect, and predictive values of these factors in Asian MESO patients should also be clarified, which would be the further research directions.

## 5. Conclusion

In this study, we constructed a model based on bone metastasis-correlated immune-related genes to predict the prognosis of patients with MESO which showed good predictive power. And we also figured out that a hub immune-related gene SDC2, regulated by specific TFs, might play an important role in bone metastasis and prognosis of MESO by regulating fraction of immune cells.

## Figures and Tables

**Table 1:** Baseline characteristics of 87 patients diagnosed with mesothelioma.

Variables	Total patients (*N* = 87)
Age (mean ± SD)	63.00 ± 9.76
Follow-up time (days)	
Mean ± SD	668.60 ± 568.34
Median (range)	527.00 (20-2790)
Gender	
Female	16 (18.39%)
Male	71 (81.61%)
Race	
Asian	1 (1.15%)
Black or African American	1 (1.15%)
White	85 (97.70%)
State	
Alive	12 (13.79%)
Dead	73 (83.91%)
Unknown	2 (2.30%)
Distant metastasis	
Yes	26 (29.89%)
No	61 (70.11%)
Bone metastasis	
Yes	4 (4.60%)
No	83 (95.40%)
Stage	
Stage I	10 (11.49%)
Stage II	16 (18.39%)
Stage III	45 (51.72)
Stage IV	16 (18.39%)
AJCC-T	
T1	14 (16.09%)
T2	26 (29.89%)
T3	32 (36.78%)
T4	13 (14.94%)
TX	2 (2.30%)
AJCC-N	
N0	44 (50.57%)
N1	10 (11.49%)
N2	26 (29.89%)
N3	3 (3.45%)
NX	4 (4.60%)
AJCC-M	
M0	57 (65.52%)
M1	3 (3.45%)
MX	27 (31.03%)

Abbreviation: AJCC: American Joint Committee on Cancer.

**(a):** 

	SDC2		TCF7L1		POLR3D		CD38		CD1A		CYP3A4		HMGCL		ACAT2		CYP27A1	
	N	T	N	T	N	T	N	T	N	T	N	T	N	T	N	T	N	T
GEPIA	NA	↑	NA	↑	NA	↑	NA	↓	NA	—	NA	↓	NA	NA	NA	↑	NA	↓
ProgGeneV2	NA	↑	NA	↑	NA	—	NA	—	NA	—	NA	NA	NA	↓	NA	↑	NA	↓
UALCAN	NA	—	NA	—	NA	↑	NA	—	NA	↑	NA	↓	NA	—	NA	↑	NA	↓
LinkedOmics	NA	—	NA	—	NA	—	NA	—	NA	—	NA	—	NA	↓	NA	—	NA	↓
UCSC Xena	NA	↑	NA	↑	NA	↑	NA	↓	NA	↑	NA	↓	NA	↓	NA	↑	NA	↓
CCLE	NA	↓	NA	↓	NA	↓	NA	↓	NA	↓	NA	↓	NA	↓	NA	—	NA	↓

**(b):** 

	PLB1		DPYS		GAD1		LTC4S		PTGS1		BCAT1		BCKDHA		BCKDHB		ACTG1	
	N	T	N	T	N	T	N	T	N	T	N	T	N	T	N	T	N	T
GEPIA	NA	↓	NA	↓	NA	↑	NA	↓	NA	↓	NA	—	NA	—	NA	↑	NA	↑
ProgGeneV2	NA	↓	NA	—	NA	↑	NA	↓	NA	↓	NA	—	NA	↓	NA	—	NA	↑
UALCAN	NA	↓	NA	↓	NA	↑	NA	—	NA	—	NA	↑	NA	—	NA	—	NA	↑
LinkedOmics	NA	—	NA	—	NA	—	NA	M	NA	—	NA	—	NA	—	NA	↑	NA	ND
UCSC Xena	NA	↓	NA	↓	NA	—	NA	↓	NA	↓	NA	—	NA	—	NA	—	NA	—
CCLE	NA	↓	NA	↓	NA	↓	NA	NA	NA	↓	NA	—	NA	—	NA	↓	NA	↑

**(c):** 

	ACTN1		Result
	N	T	
GEPIA	NA	↑	SDC2, TCF7L1, POLR3D, ACAT2, GAD1, BCKDHB, ACTG1, and ACTN1 were highly expressed in MESO (Figure S1 C, F, I; Figure S3 B, F, I, J, K). CD38, CYP3A4, CYP27A1, PLB1, DPYS, LTC4S, and PTGS1 were lowly expressed in GEPIA (Figure S2 C, Figure S3 A, C, D, E, G, H).
ProgGeneV2	NA	↑	In ProgGeneV2, SDC2, TCF7L1, ACAT2, GAD1, ACTG1, and ACTN1 were expressed highly in MESO (Figure S1 E, H; Figure S4 B, E, I, J). HMGCL, CYP27A1, PLB1, LTC4S, PTGS1, and BCKDHA were expressed lowly in MESO (Figure S4 A, C, D, F, G, H).
UALCAN	NA	↑	In UALCAN, POLR3D, CD1A, ACAT2, GAD1, BCAT1, ACTG1, and ACTN1 were highly expressed in MESO (Figure S1 K; Figure S2 D; Figure S5 B, F, G, H, I). CYP3A4, CYP27A1, PLB1, and DPYS were lowly expressed in MESO (Figure S5 A, C, D, E).
LinkedOmics	NA	—	In LinkedOmics, BCKDHB was highly expressed in MESO (Figure S6 F). HMGCL and CYP27A1 were lowly expressed in MESO (Figure S6 D, E).
UCSC Xena	NA	↑	In UCSC Xena, SDC2, TCF7L1, POLR3D, CD1A, ACAT2, and ACTN1 were highly expressed in MESO (Figure S1 D, G, J; Figure S2 E; Figure S7 C, I). CD38, CYP3A4, HMGCL, CYP27A1, PLB1, DPYS, LTC4S, and PTGS1 were lowly expressed in MESO (Figure S2 B; Figure S7 A, B, D, E, F, G, H).
CCLE	NA	—	In CCLE, ACTG1 was highly expressed in MESO and SDC2, TCF7L1, POLR3D, CD38, CD1A, CYP3A4, HMGCL, CYP27A1, PLB1, DPYS, GAD1, PTGS1, and BCKDHB were lowly expressed in MESO (Figure S8 B).

“N” was defined as normal; “T” was defined as thyroid carcinoma; “↑” was defined as a significantly high-expressed gene; “↓” was defined as a significantly low-expressed gene; “NA” was defined as “not available”; “ND” was defined as “not detached”; “-” was defined as a gene with no significant difference in expression. Abbreviations: MESO: mesothelioma; GEPIA: Gene Expression Profiling Interactive Analysis; CCLE: Cancer Cell Line Encyclopedia.

## Data Availability

The datasets generated and/or analyzed during the current study are available in the Supplementary Material and TCGA-MESO program (https://portal.gdc.cancer.gov).

## Abbreviations

KEGG: Kyoto Encyclopedia of Genes and Genomes
ND: Not detected.

## Supplementary Materials

Supplementary Materials Table S1: biomarkers used in external validation. Figure S1: external validation of key characters. (A) Overview of 60 biomarkers brought into external validation using String database; (B) in UALCAN, SDC2 was differently expressed in various cancers; (C, D, E) SDC2 was significantly associated with overall survival (OS) of patients with MESO in GEPIA (C), UCSC (D), and ProgGeneV2 (E); (F, G, H) TCF7L1 was significantly associated with OS in GEPIA (F), UCSC (G), and ProgGeneV2 (H); (I, J, K) POLR3D was significantly correlated with OS of MESO in GEPIA (I), UCSC (J), and UALCAN (K). Figure S2: results of immune cell markers. Plasma cell marker CD38 was significantly associated with OS in in UCSC (A) and GEPIA (B) and was correlated with distant metastasis in LinkedOmics (C). CD1A, cell marker of dendritic cells, DCs, and iDCs, was correlated with OS in UCSC (D) and UALCAN (E). Figure S3: validation of pathway biomarkers in MESO patients using the GEPIA database. In GEPIA, CYP3A4 (A), ACAT2 (B), CYP27A1 (C), PLB1 (D), DPYS (E), GAD1 (F), LTC4S (G), PTGS1 (H), BCKDHB (I), ACTG1 (J), and ACTN1 (K) were associated with OS on tissue level in GEPIA. Figure S4: results of external validation using ProgGeneV2. HMGCL (A), ACAT2(B), CYP27A1 (C), PLB1 (D), GAD1 (E), LTC4S (F), PTGS1 (G), BCKDHA (H), ACTG1 (I), and ACTN1 (J) were significantly associated with OS of MESO patients. Figure S5: external validation results using UALCAN. In UALCAN, CYP3A4 (A), ACAT2 (B), CYP27A1 (C), PLB1 (D), DPYS (E), GAD1 (F), BCAT1 (G), ACTG1 (H), and ACTN1 (I) were correlated with OS significantly. Figure S6: results of external validation using LinkedOmics. (A) Summary of genes positively associated with OS in MESO; (B) summary of genes negatively associated with OS in MESO; (C) evaluation of the correlation between genes and OS; (D, E, F) in LinkedOmics, HMGCL (D) and LTC4S (E) and BCKDHB (F) were significantly associated with OS; (G) HMGCL was significantly associated with distant metastasis; (H) ACTN1 was significantly associated with distant metastasis. Figure S7: results of external validation using the UCSC Xena database. In UCSC, CYP3A4 (A), HMGCL (B), ACAT2 (C), CYP27A1 (D), PLB1 (E), DPYS (F), LTC4S (G), PTGS1 (H), and ACTN1 (I) were significantly associated with OS of MESO patients. Figure S8: (A) gene modification information of key factors derived from cBioPortal database; (B) expression level of key genes in pleura based on data from the CCLE database.
