# Supplementary material for: Prognostic Bone Metastasis-Associated Immune-Related Genes Regulated by Transcription Factors in Mesothelioma
Source: Biomed Res Int. 2022 Jan 27;2022:9940566. doi: 10.1155/2022/9940566 (PMC8813231; doi:10.1155/2022/9940566)
Supplement: Supplementary Materials — Table S1: biomarkers used in external validation. Figure S1: external validation of key characters. (A) Overview of 60 biomarkers brought into external validation using String database; (B) in UALCAN, SDC2 was differently expressed in various cancers; (C, D, E) SDC2 was significantly associated with overall survival (OS) of patients with MESO in GEPIA (C), UCSC (D), and ProgGeneV2 (E); (F, G, H) TCF7L1 was significantly associated with OS in GEPIA (F), UCSC (G), and ProgGeneV2 (H); (I, J, K) POLR3D was significantly correlated with OS of MESO in GEPIA (I), UCSC (J), and UALCAN (K). Figure S2: results of immune cell markers. Plasma cell marker CD38 was significantly associated with OS in in UCSC (A) and GEPIA (B) and was correlated with distant metastasis in LinkedOmics (C). CD1A, cell marker of dendritic cells, DCs, and iDCs, was correlated with OS in UCSC (D) and UALCAN (E). Figure S3: validation of pathway biomarkers in MESO patients using the GEPIA database. In GEPIA, CYP3A4 (A), ACAT2 (B), CYP27A1 (C), PLB1 (D), DPYS (E), GAD1 (F), LTC4S (G), PTGS1 (H), BCKDHB (I), ACTG1 (J), and ACTN1 (K) were associated with OS on tissue level in GEPIA. Figure S4: results of external validation using ProgGeneV2. HMGCL (A), ACAT2(B), CYP27A1 (C), PLB1 (D), GAD1 (E), LTC4S (F), PTGS1 (G), BCKDHA (H), ACTG1 (I), and ACTN1 (J) were significantly associated with OS of MESO patients. Figure S5: external validation results using UALCAN. In UALCAN, CYP3A4 (A), ACAT2 (B), CYP27A1 (C), PLB1 (D), DPYS (E), GAD1 (F), BCAT1 (G), ACTG1 (H), and ACTN1 (I) were correlated with OS significantly. Figure S6: results of external validation using LinkedOmics. (A) Summary of genes positively associated with OS in MESO; (B) summary of genes negatively associated with OS in MESO; (C) evaluation of the correlation between genes and OS; (D, E, F) in LinkedOmics, HMGCL (D) and LTC4S (E) and BCKDHB (F) were significantly associated with OS; (G) HMGCL was significantly associated with distant metastasis; [file 9940566.f1.docx]

**Table S1 Biomarkers used in external validation**

|  | | **Biomarkers** | | | | |
| --- | --- | --- | --- | --- | --- | --- |
|  | |  | | | | |
| **Key gene** | | SDC2 |  |  |  |  |
| **Transcription factors** | | RCOR1 | TCF7L1 | POLR3D | NR2F2 |  |
| **Immune cells** |  |  |  |  |  |  |
|  | Plasma cells | CD38 | CD19 | IGHA2 |  |  |
|  | Macrophages M2 | CD163 | CD206 | CCL18 |  |  |
|  | Dendritic cells resting | CD83 | CD11C | CD1a | CD209 | CLEC4C |
|  | DCs | CD83 | CD11C | CD1a | CD209 | CLEC4C |
|  | iDCs | CD83 | CD11C | CD1a | CD209 | CLEC4C |
|  | NK_cells | IOT-10 | CD16 | CD56 | CD3 |  |
|  | Cytolytic_activity | ND | ND | ND | ND | ND |
|  | MHC_class_I | ND | ND | ND | ND | ND |
|  | Parainflammation | ND | ND | ND | ND | ND |
|  | Type_I_IFN_Reponse | ND | ND | ND | ND | ND |
|  | Type_II_IFN_Reponse | ND | ND | ND | ND | ND |
| **KEGG pathways** |  |  |  |  |  |  |
|  | Metabolism of xenobiotics by cytochrome P450 | CYP3A4 | CYP2C9 | UGT1A9 | UGT2B7 | CYP1A2 |
|  | Retinol metabolism | CYP3A4 | CYP2C9 | UGT1A9 | UGT2B7 | CYP1A2 |
|  | Drug metabolism cytochrome P450 | CYP3A4 | CYP2C9 | UGT1A9 | UGT2B7 | CYP1A2 |
|  | Butanoate metabolism | HMGCL | HMGCS1 | HMGCS2 | ACAT1 | ACAT2 |
|  | Primary bile acid biosynthesis | CYP8B1 | CYP27A1 | AKR1D1 | SLC27A5 | HSD3B7 |
|  | Linoleic acid metabolism | PLA2G4B | PLAAT3 | PLA2G4E | PLB1 | PLA2G4F |
|  | Beta alanine metabolism | DPYD | DPYS | UPB1 | GAD1 | GAD2 |
|  | Arachidonic acid metabolism | ALOX5 | LTC4S | PTGS1 | PTGS2 | ALOX5AP |
|  | Valine leucine and isoleucine degradation | BCAT1 | BCAT2 | BCKDHA | BCKDHB | DBT |
|  | Regulation of actin cytoskeleton | ACTB | ACTG1 | ACTN1 | APC | RHOA |

**Abbreviations:** KEGG, Kyoto Encyclopedia of Genes and Genomes.

**Note:** “ND” was defined as not detected.


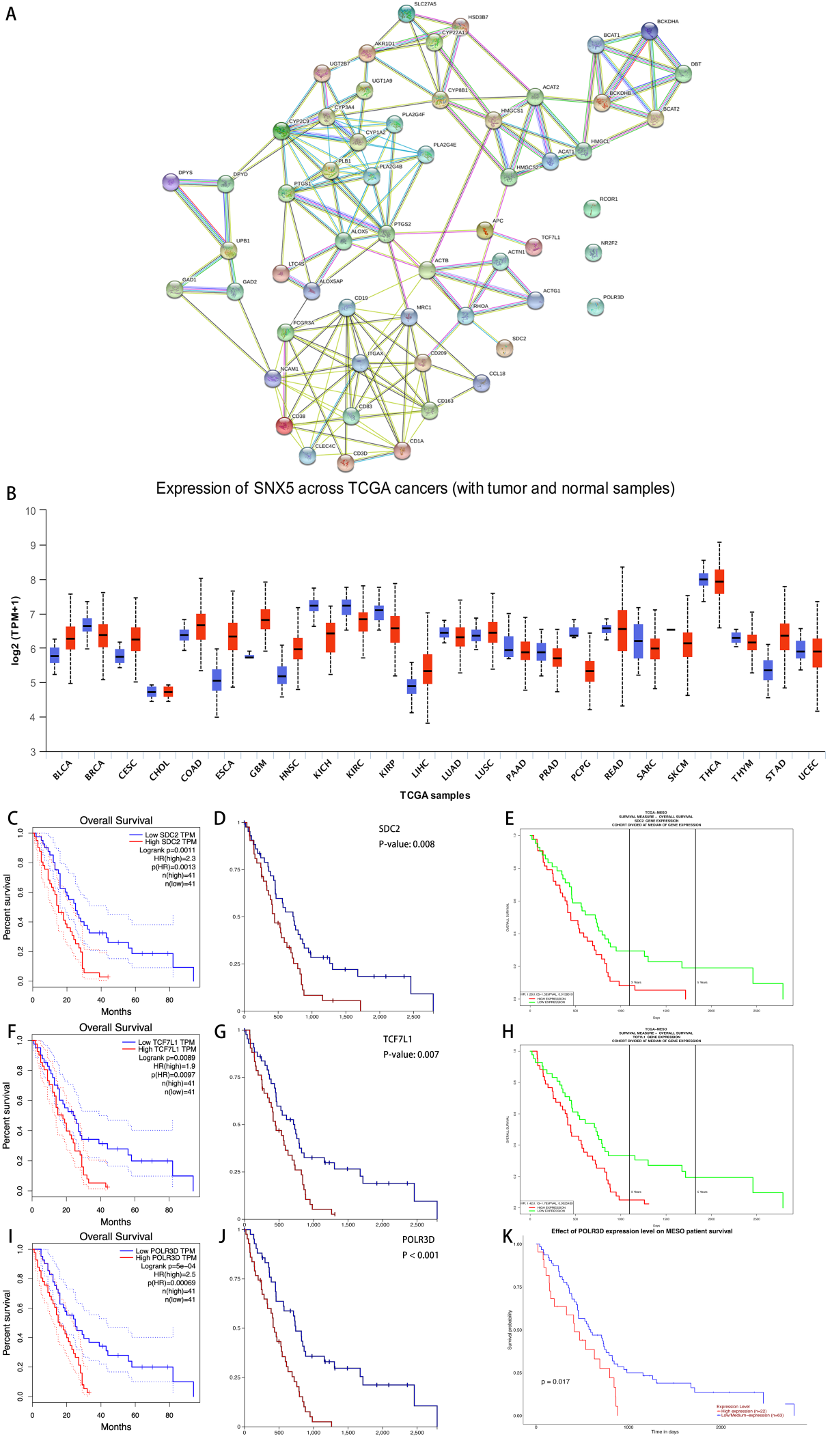


Figure S1 External validation of key characters. (A) Overview of 60 biomarkers brought into external validation using String database; (B) In UALCAN, SDC2 was differently expressed in various cancers; (C, D, E) SDC2 was significantly associated with overall survival (OS) of patients with MESO in GEPIA (C), UCSC (D), and ProgGeneV2 (E); (F, G, H) TCF7L1 was significantly associated with OS in GEPIA (F), UCSC (G), and ProgGeneV2 (H); (I, J, K) POLR3D was significantly correlated with OS of MESO in GEPIA (I), UCSC (J) and UALCAN (K).


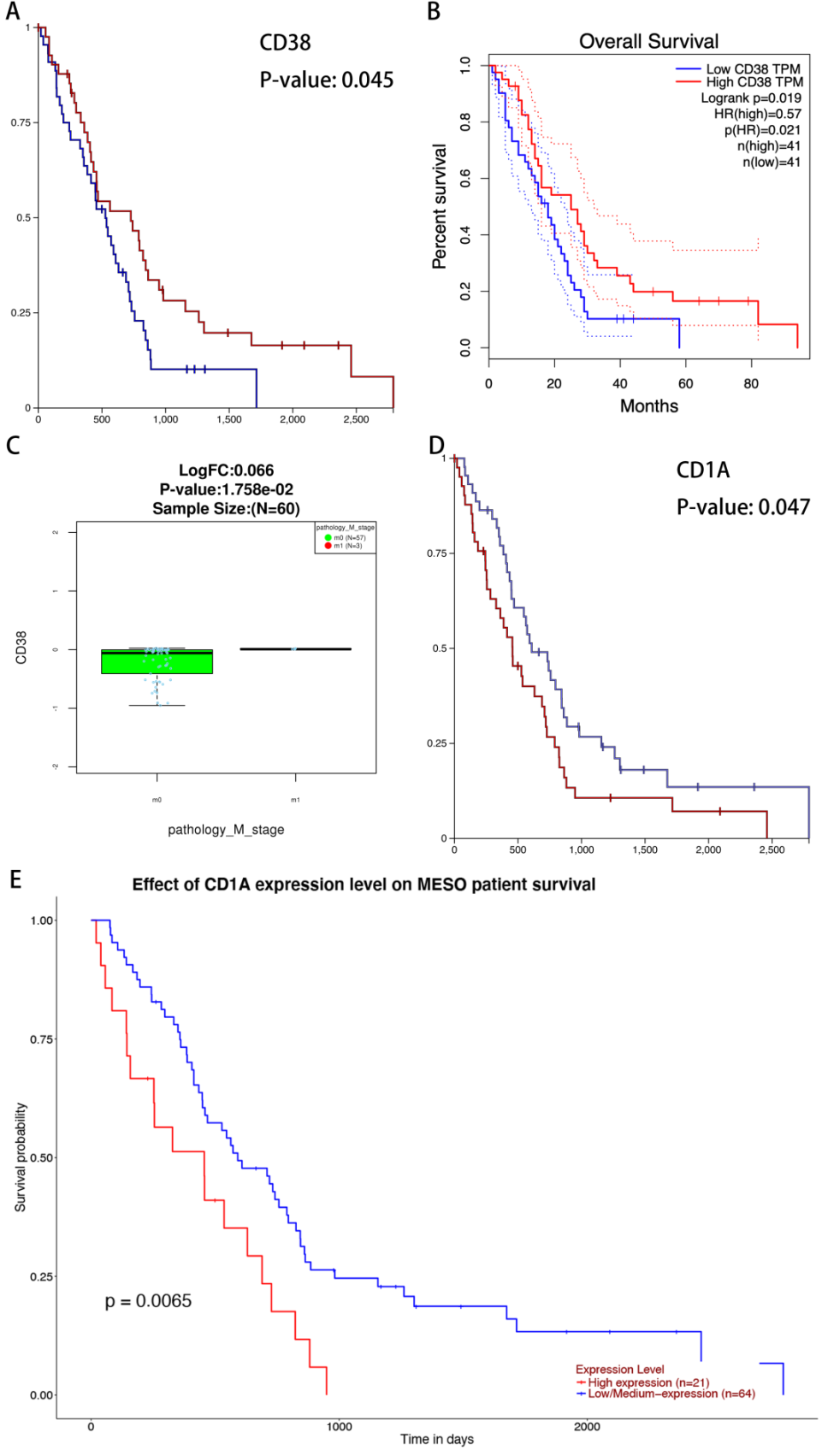


Figure S2 Results of immune cell markers. Plasma cell marker CD38 was significantly associated with OS in in UCSC (A) and GEPIA (B) and was correlated with distant metastasis in LinkedOmics (C). CD1A, cell marker of dendritic cells, DCs and iDCs, was correlated with OS in UCSC (D) and UALCAN (E).


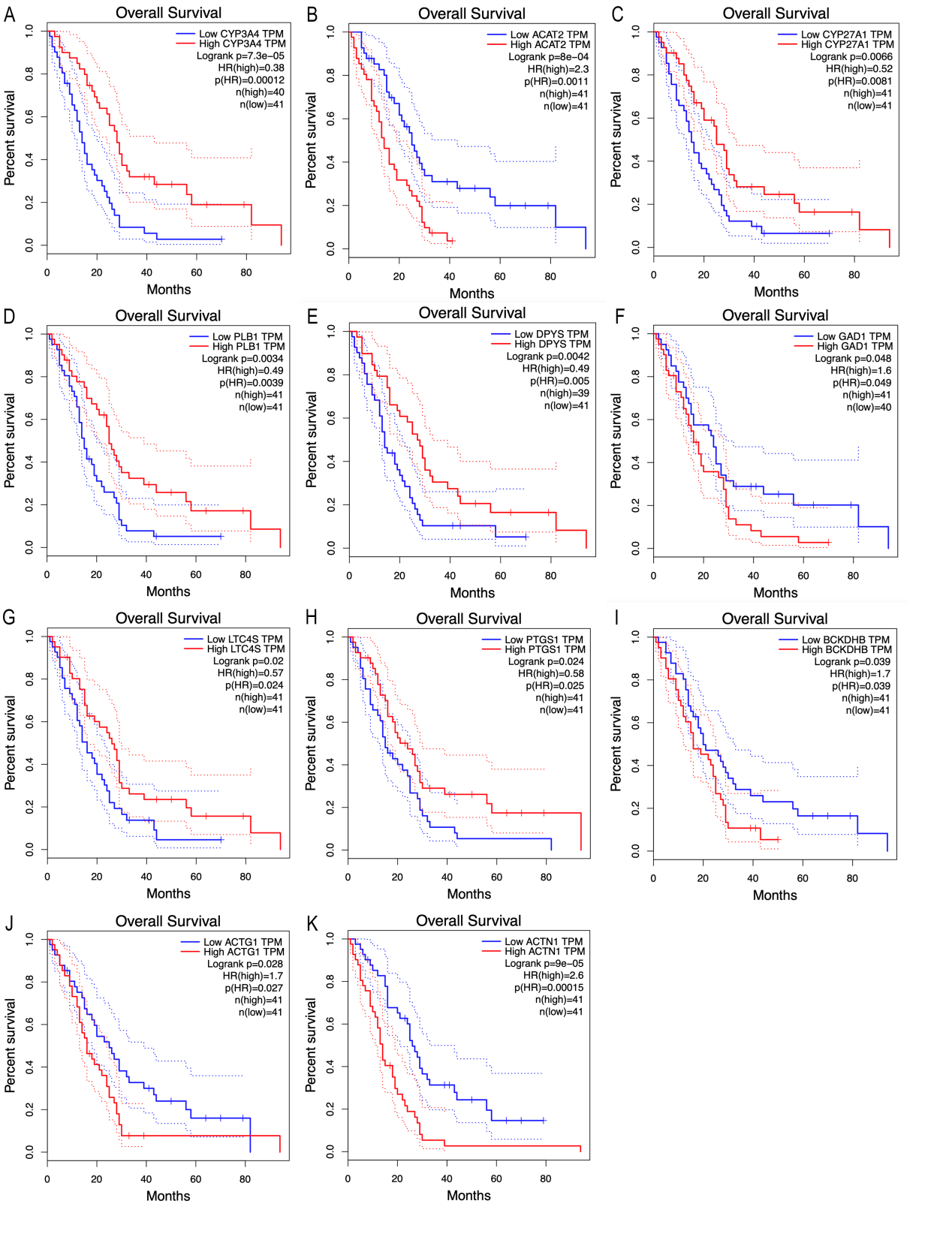


Figure S3 Validation of pathway biomarkers in MESO patients using GEPIA database. In GEPIA, CYP3A4 (A), ACAT2 (B), CYP27A1 (C), PLB1 (D), DPYS (E), GAD1 (F), LTC4S (G), PTGS1 (H), BCKDHB (I), ACTG1 (J) and ACTN1 (K) were associated with OS on tissue level in GEPIA.


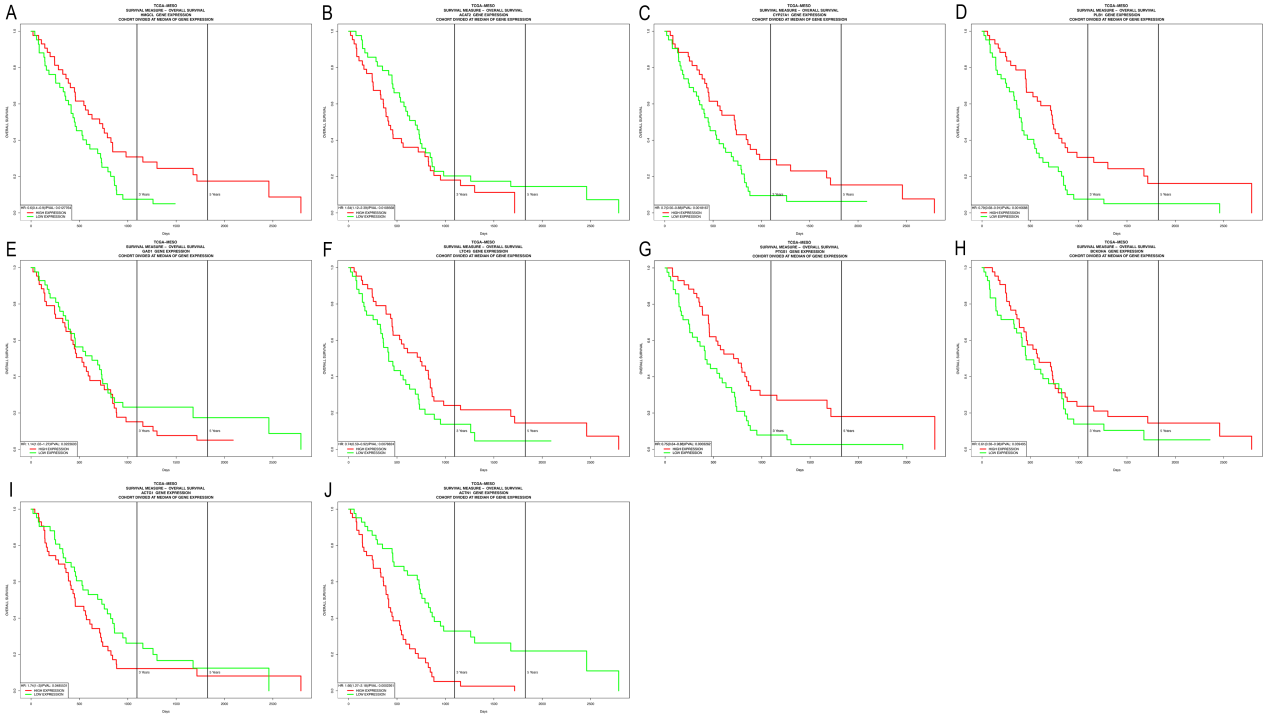


Figure S4 Results of external validation using ProgGeneV2. HMGCL (A), ACAT2(B), CYP27A1 (C), PLB1 (D), GAD1 (E), LTC4S (F), PTGS1 (G), BCKDHA (H), ACTG1 (I) and ACTN1 (J) were significantly associated with OS of MESO patients.


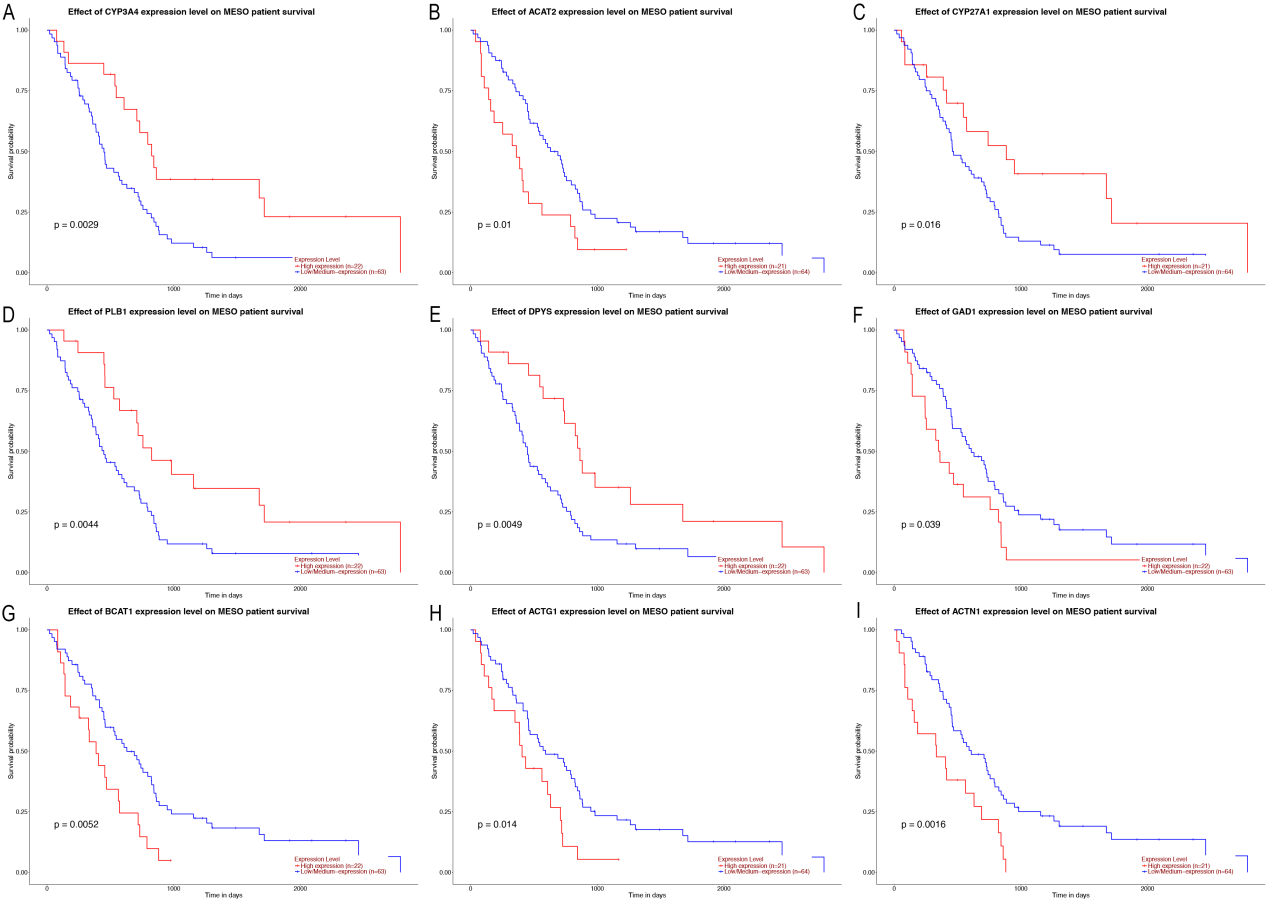


Figure S5 External validation results using UALCAN. In UALCAN, CYP3A4 (A), ACAT2 (B), CYP27A1 (C), PLB1 (D), DPYS (E), GAD1 (F), BCAT1 (G), ACTG1 (H) and ACTN1 (I) were correlated with OS significantly.


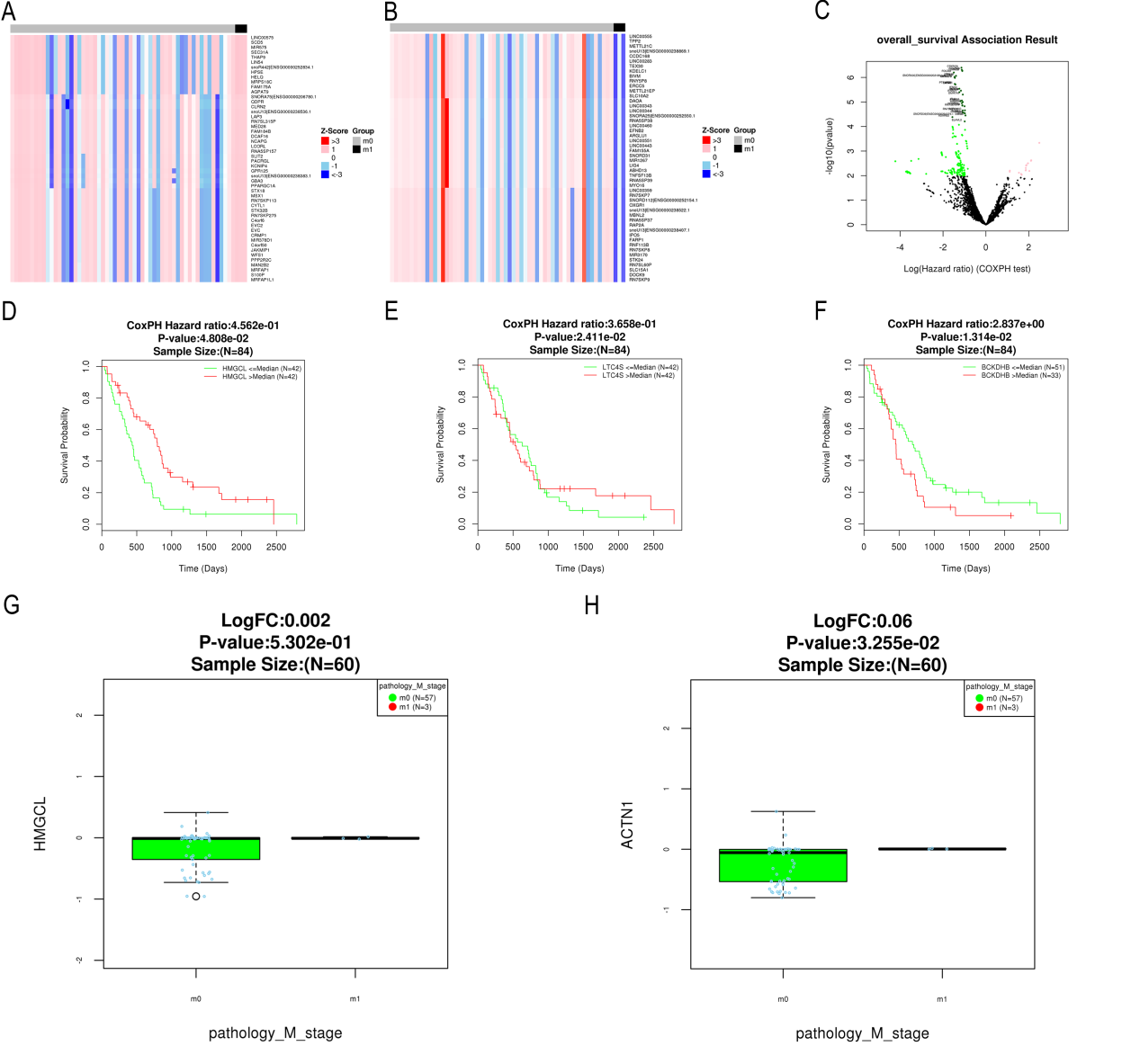


Figure S6 Results of external validation using LinkedOmics. (A) Summary of genes positively associated with OS in MESO; (B) Summary of genes negatively associated with OS in MESO; (C) Evaluation of the correlation between genes and OS; (D, E, F) In LinkedOmics, HMGCL (D) and LTC4S (E) and BCKDHB (F) were significantly associated with OS; (G) HMGCL was significantly associated with distant metastasis; (H) ACTN1 was significantly associated with distant metastasis.


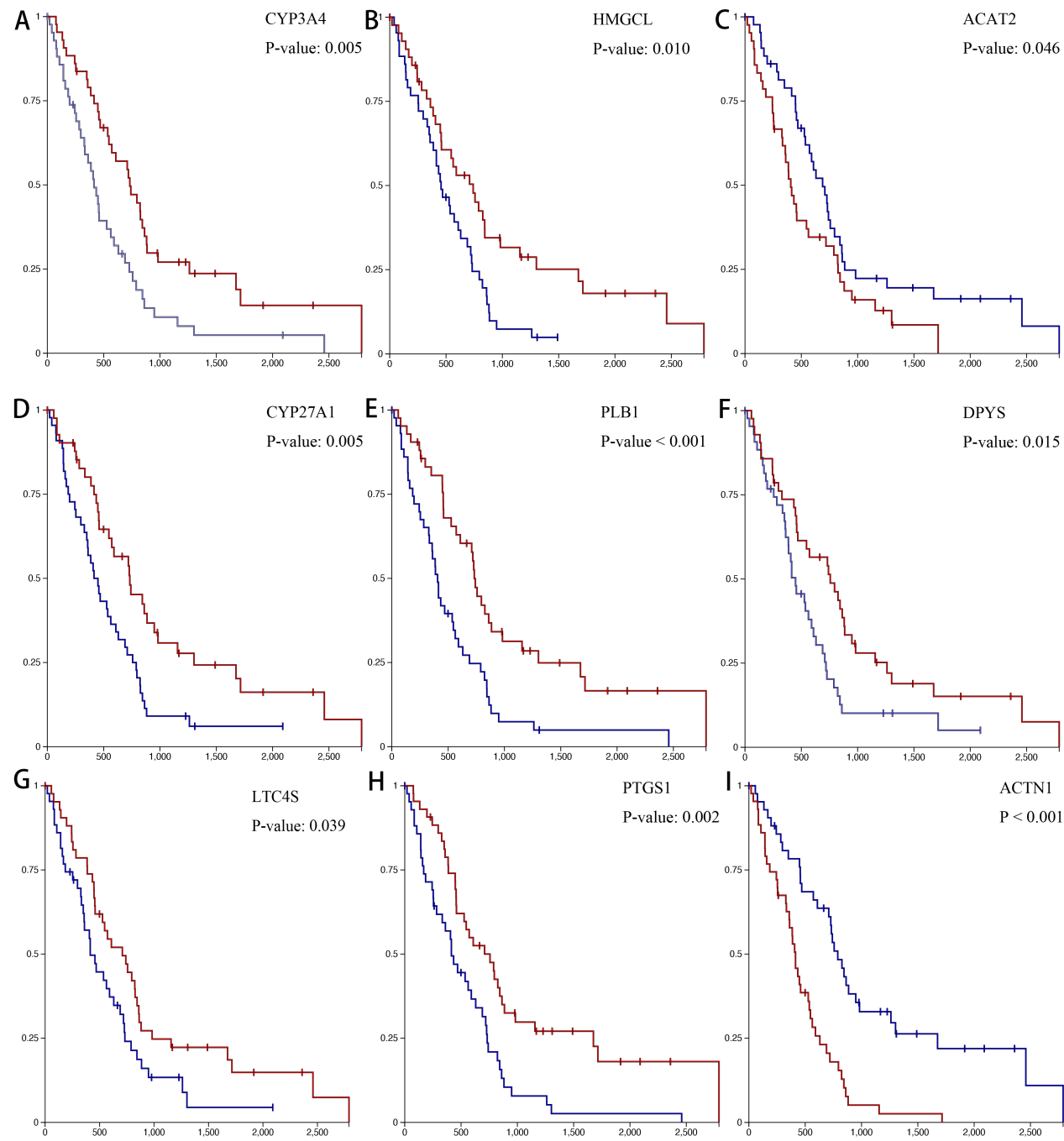


Figure S7 Results of external validation using UCSC Xena database. In UCSC, CYP3A4 (A), HMGCL (B), ACAT2 (C), CYP27A1 (D), PLB1 (E), DPYS (F), LTC4S (G), PTGS1 (H) and ACTN1 (I) were significantly associated with OS of MESO patients.


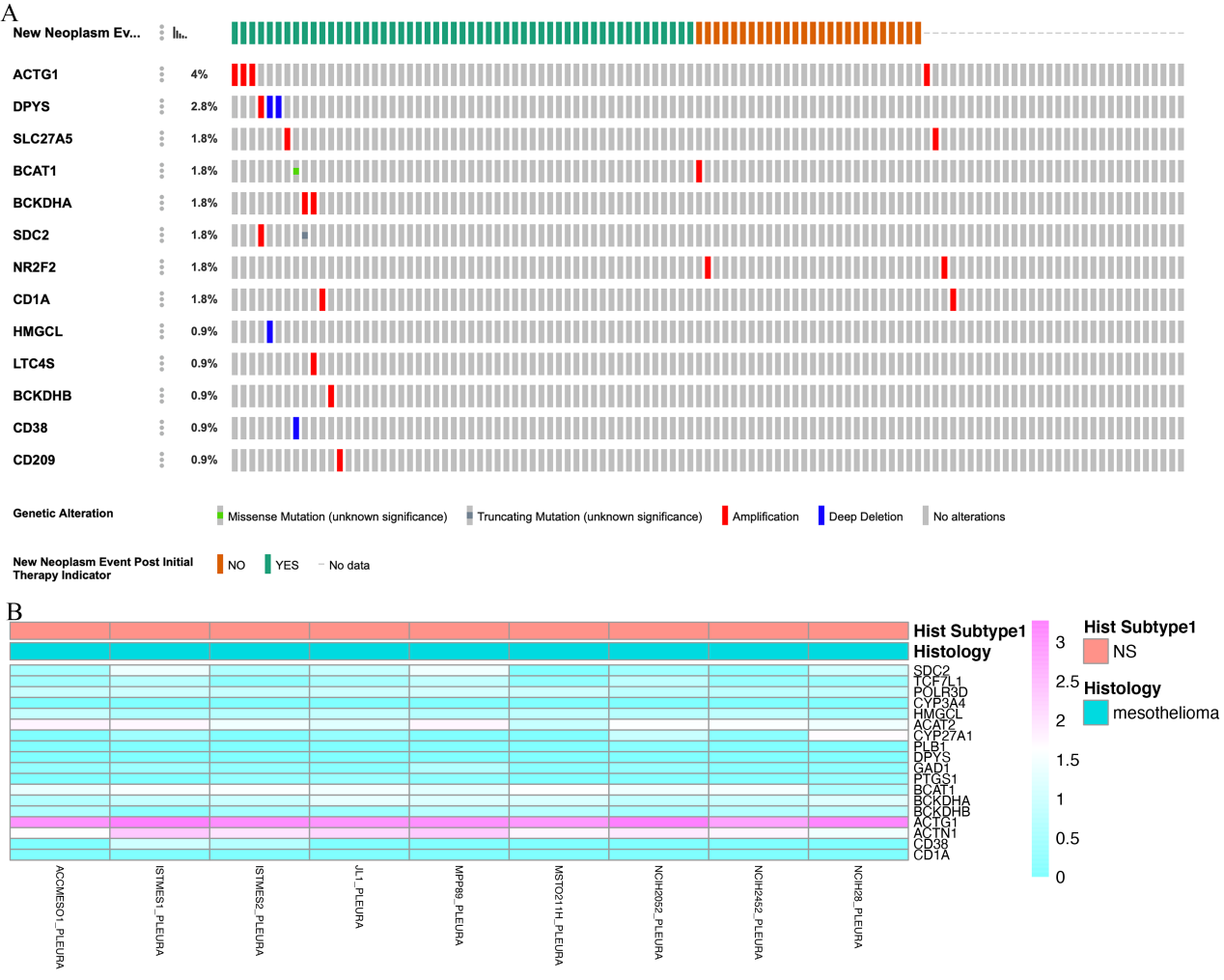


Figure S8 (A) Gene modification information of key factors derived from cBioPortal database; (B) Expression level of key genes in pleura based on data from CCLE database.
